# Evaluation of an eHealth Intervention in Chronic Care for Frail Older People: Why Adherence is the First Target

**DOI:** 10.2196/jmir.3057

**Published:** 2014-06-23

**Authors:** Peter Makai, Marieke Perry, Sarah HM Robben, Henk J Schers, Maud M Heinen, Marcel GM Olde Rikkert, René F Melis

**Affiliations:** ^1^Radboud University Medical CenterDepartment of GeriatricsNijmegenNetherlands; ^2^Jeroen Bosch HospitalDepartment of Internal MedicineHertogenboschNetherlands; ^3^Radboud University Medical CenterDepartment of Primary and Community CareNijmegenNetherlands; ^4^Radboud Univesrity Medical CenterScientific Institute for Quality in HealthcareNijmegenNetherlands

**Keywords:** eHealth, frail elderly, care coordination, chronic care

## Abstract

**Background:**

Older people suffering from frailty often receive fragmented chronic care from multiple professionals. According to the literature, there is an urgent need for coordination of care.

**Objective:**

The objective of this study was to investigate the effectiveness of an online health community (OHC) intervention for older people with frailty aimed at facilitating multidisciplinary communication.

**Methods:**

The design was a controlled before-after study with 12 months follow-up in 11 family practices in the eastern part of the Netherlands. Participants consisted of frail older people living in the community requiring multidisciplinary (long-term) care. The intervention used was the health and welfare portal (ZWIP): an OHC for frail elderly patients, their informal caregivers and professionals. ZWIP contains a secure messaging system supplemented by a shared electronic health record. Primary outcomes were scores on the Instrumental Activities of Daily Living scale (IADL), mental health, and social activity limitations.

**Results:**

There were 290 patients in the intervention group and 392 in the control group. Of these, 76/290 (26.2%) in the intervention group actively used ZWIP. After 12 months follow-up, we observed no significant improvement on primary patient outcomes. ADL improved in the intervention group with a standardized score of 0.21 (*P*=.27); IADL improved with 0.50 points, *P*=.64.

**Conclusions:**

Only a small percentage of frail elderly people in the study intensively used ZWIP, our newly developed and innovative eHealth tool. The use of this OHC did not significantly improve patient outcomes. This was most likely due to the limited use of the OHC, and a relatively short follow-up time. Increasing actual use of eHealth intervention seems a precondition for large-scale evaluation, and earlier adoption before frailty develops may improve later use and effectiveness of ZWIP.

## Introduction

Chronic care for frail older people is fragmented, with involvement from a large and constantly changing group of professionals who are frequently unaware that they provide care to the same patient [1]. Such professionals include home care professionals, general practitioners (GPs), clinicians, physiotherapists, and case managers dedicated to long-term care of the patients in the community. Frail elderly often suffer from comorbidities, which results in care by multiple health care professionals [2]. Therefore lack of communication between professionals leads to a fragmented and ineffective health care delivery for frail elderly [3]. To reduce fragmentation and promote continuity of care, better coordination and communication between professionals and with patients is necessary. Online health communities (OHCs) have been recognized as an effective mechanism for supporting continuous care for frail older people [4], allowing better coordination and more efficient communication with patients and among professionals. OHCs consist of Internet-based platforms that unite groups of individuals with a shared goal or similar interest, including both professionals and patients [5]. The main strength of OHCs is that they allow communication between people who would not have met each other otherwise [5]. Thus, OHCs are particularly suited for improving the coordination of care for frail elderly who have multiple professional caregivers. For this purpose, we developed and evaluated the Health and Welfare Information Portal (Zorg en Welzijns Informatie Portaal, ZWIP, in Dutch) [1,6] on its effectiveness.

## Methods

### Intervention

**Figure 1:**
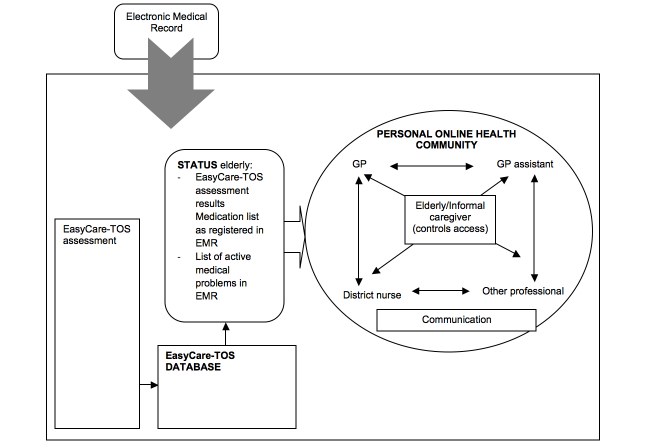
A conceptual model of the ZWIP.

ZWIP is an OHC [5] that aims to facilitate communication for patients, their informal caregivers, and their professionals. ZWIP contains a secure messaging system supplemented by a shared electronic health record. All messages shared in a patient’s ZWIP are visible for all users, thus stimulating involvement of and discussion between patients and a team of health professionals. All informal caregivers and health care professionals have access to the electronic health record. To ensure confidentiality, professionals can participate in a patient’s personal care network in ZWIP only at the invitation of the patient. Patients who were not able to manage their own ZWIP account could appoint an informal caregiver to act on their behalf. Figure 1 demonstrates the conceptual model underlying ZWIP, and the video in Multimedia Appendix 1 illustrates the use of ZWIP by a patient and an informal caregiver.

### Development and Implementation

The development of ZWIP and the process of implementation have been described elsewhere [1,6]. In brief, ZWIP was developed using intervention mapping [7], a stepwise approach for the systematic development of interventions informed by both evidence and theory [1]. Main steps of intervention mapping for ZWIP were (1) needs assessment in frail elderly, (2) developing program objectives, (3) selecting theory informed intervention methods and strategies, (4) creating and pilot testing program components, (5) planning program adoption and implementation, and (6) planning for evaluation [1]. Theoretically, ZWIP was based on social cognitive theory [8], with special attention paid to improving self-efficacy, the belief people have in their ability to complete tasks and achieve specific goals [9]. Following the steps of intervention mapping and as suggested in the guideline on development and evaluation of complex interventions [8], the ZWIP was piloted by 2 frail elderly and 7 professionals, including one GP. Furthermore, newly developed elements of ZWIP were regularly piloted by similar user panels.

To enhance implementation of the ZWIP, we used several strategies for professionals such as a continuing medical education (CME) accredited education program based on active learning theory [10], direct experience, and modeling [1]. Additionally, drawing from organization theory, we installed a telephonic helpdesk and provided e-coaching and financial compensation to support the uptake of ZWIP by professionals [7,11]. To facilitate the use of ZWIP among elderly patients, a number of approaches were used: flyers were distributed in the primary care centers, a hard-copy version of ZWIP was provided in order for patients to familiarize themselves with ZWIP, coaching on the use of ZWIP was made available, involvement of informal caregivers was encouraged, and the GPs actively advocated the use of ZWIP, thus drawing on modeling, guided practice, and tailoring support for use of the intervention [1,6]. During the implementation phase, we designated one key person in each family practice who coordinated implementation activities and helped colleagues with questions [6].

### Inclusion and Design

Between July 2010 and July 2011, frail older patients were included in an observational, controlled before-after study with 12 months follow-up to investigate ZWIP’s effects on patient outcomes. Participating primary care centers were recruited from the university primary care network around the city of Nijmegen, the Netherlands. These centers identified their frail older people using the EASYcare Two-step Older person Screening (TOS) instrument [12]. Therefore, both intervention and control practices had to implement an identification scheme and redesign care for their frail elderly. Interventions centers were selected based on willingness to participate in ZWIP, whereas control practices were selected from a separate project: the EASYcare-TOS validation study [13]{van Kempen, 2013 #7718}. Frail status as determined by the EASYcare-TOS was the only inclusion criterion for included patients. Patients in the intervention group patients needed to agree to the creation of a ZWIP account. No exclusion criteria were specified.

All measurements were performed by trained nurses in the patients’ homes, using a face-to-face questionnaire at baseline and at follow-up. The study was exempt from ethics review by the local ethics committee because of its observational nature and nonintrusive data collection. Nevertheless, oral informed consent was obtained to analyze the data during data collection.

### Outcomes

Primary outcomes were Activities of Daily Living (ADL) as measured by the Katz index [14], combined ADL and Instrumental Activities of Daily Living (IADL) as measured by the Katz-15, a combined measure of the ADL and Lawton-index [14,15], SF-36 mental health and social activity limitation dimensions [16]. The Katz index consists of yes or no responses on ADL items such as bathing or dressing. ADL scores range from 0-6 with higher scores indicating higher dependency. The Katz-15 consists of yes or no responses on ADL and additional IADL items such as using the telephone and managing money [14]. The Katz-15 scores range from 0-15 with higher scores indicating more limitations. Both scales are established in the literature and have adequate reliability and validity [17]. The SF-36 mental health dimension, consisting of the following subscales: happy, calm, blue, down, nervous scoring from 0-5 with higher numbers indicating a higher score. The scores were summed into a summary score ranging from 0-100, with 100 indicating full mental health, and 0 low mental health [16]. To assess differences in social activity limitations, the social activity limitation item from the SF-36 was used [16]. This item measures the frequency in which respondents experienced social activity limitations due to health. The item used in this current study is scored from 0 (none of the time) to 5 (all of the time). The various SF-36 subscales have excellent reliability and validity [17]. Secondary outcomes were several self-developed scales of patient satisfaction and GPs’ subjective experience with care coordination. Patient satisfaction items were scored on a 5-point Likert scale ranging from 1 (way too little or way too much) to 5 (optimal), similar to this article [18]. GP experience with coordination of care was scored between 1 (uncoordinated) to 10 (optimal coordination). Important covariates were measured including a frailty index based on the accumulation of deficits concept [19,20]. The frailty index is the number of deficits present divided by a total possible number of deficits [2]. As such, the frailty index can account for all kinds of health-related imbalances between the intervention and control group and provides an accurate measure of individuals’ frailty.

### Analysis

For comparing baseline characteristics, chi-square tests were used to compare nominal variables, and *t* tests were used for normally distributed continuous variables. Effects were determined using linear mixed models within a highly efficient analysis of covariance (ANCOVA) framework [21] to allow for clustering within a primary care center. Adjustments were made for frailty status and centered baseline status of the outcome variable and additional covariates with baseline imbalance. All analyses were performed with SAS 9.2.

## Results

Overall, 290/622 (46.6%) of all frail persons identified within 11 practices participated in the intervention group. From 6 practices 392 frail older people participated in the control group. At 12-month follow-up, in the intervention group 179/290 (61.7% of original) patients provided data at follow-up, versus 270/392 (68.8% of original) patients in the control group. At baseline, participants in the intervention group were more likely to have completed primary education only, have more informal caregivers, and have higher complexity of care compared to the participants in the control group. Further, participants in the intervention groups also had a higher average frailty index score, and GPs had lower experience with coordination of care (Table 1).

One quarter 45/117 (25%) of all patients in the intervention group used ZWIP at least once a month during a period of 12 months. Controlling for frailty and other unbalanced baseline characteristics, we found no significant differences in primary patient outcomes (Table 2). Change in coordination of care as reported by GPs improved in the control group.

**Table 1:** Demographic and care-related characteristics in the intervention and control group.

Demographic and care-related characteristics	Category	Total	Intervention ZWIP, n=179, n (%)	Control regular care, n=270, n (%)	*P* value^a^
Sex, n (%)	Female	284	117 (65.4)	167 (61.8)	.45
Age, mean (SD)		449	81.69 (5.38)	81.32 (5.72)	.49
**Education, n (%)**					
	Primary or less than primary education	67	30 (19.1)	37 (14.8)	<.01
	Secondary education	339	123 (76.5)	216 (82.1)	
	University/tertiary education	15	7 (4.4)	8 (3.1)	
**Marital status, n (%)**					
	Married	196	80 (45.2)	116 (44.3)	.43
	Divorced	28	7 (3.4)	21 (7.4)	
	Widow/widower/partner deceased	199	79 (44.3)	120 (44.1)	
	Unmarried	24	12 (6.7)	12 (4.2)	
Informal caregiver, n (%)	Available	253	147 (82.6)	106 (39.7)	<.01^a^
Living independently, n (%)	Yes	230	86 (50.3)	144 (53.0)	.46
**Complexity of care, n (%)**					
	One professional	63	12 (6.8)	51 (18.7)	<.01^a^
	2 or 3 professionals	311	128 (71.9)	183 (67.4)	
	>3 professionals	74	38 (21.3)	36 (13.9)	
Frailty index, mean (SD)		447	0.29 (0.07)	0.27 (0.07)	.02^a^
Multimorbidity, mean (SD)		447	1.70 (1.22)	1.73 (1.35)	.78
GP experience with coordination of care around the patient, mean (SD)		449	5.92 (2.36)	6.76 (3.45)	<.01^a^

^a^2-sided chi-square for discrete and *t* tests for continuous variables.

**Table 2:** Change in outcomes by 12 months application of the ZWIP Web-based tool for patient-professional and interprofessional communication.

Variable	Total	Intervention ZWIP baseline	Intervention ZWIP follow-up	Control baseline	Control follow-up	Standardized difference between study groups	*P* value (mixed model)
	N	mean (95% CI)	mean (95% CI)	mean (95% CI)	mean (95% CI)	mean, (95% CI)	
Katz ADL	442	1.09 (0.91-1.27)	1.35 (1.14-1.56)	0.85 (0.72-0.98)	1.02 (0.86-1.18)	0.21 (-0.17-0.59)	.27
Katz-15	442	5.08 (4.73-5.44)	5.76 (5.32-6.21)	4.24 (3.92-4.57)	4.93 (4.58-5.28)	0.50 (-1.59-2.60)	.64
SF-36 mental health	440	76.30 (74.32-78.28)	74.59 (72.83-76.36)	76.27 (74.77-77.77)	79.06 (77.36-80.75)	-8.34 (-17.02-0.34)	.06
SF-36 social	436	1.44 (1.23-1.64)	1.20 (1.01-1.39)	0.87 (0.73-1.01)	0.93 (0.79-1.08)	0.84 (-0.78-2.45)	.31
Patient experience with coordination of care	303	4.66 (4.50-4.83)	4.78 (4.65-4.91)	4.59 (4.44-4.75)	4.77 (4.67-4.88)	-0.25 (-0.99-0.49)	.58
Patient experience with co-decision making	399	3.51 (3.41-3.61)	4.86 (4.77-4.95)	3.63 (3.54-3.72)	4.73 (4.62-4.83)	0.16 (-0.40-0.71)	.64
Patient preferences for influence	414	3.59 (3.42-3.75)	3.59 (3.42-3.76)	3.22 (3.08-3.36)	3.33 (3.20-3.45)	0.36 (-1.28-1.99)	.08
Patient knowledge of providers (health and social)	432	3.51 (3.41-3.61)	3.57 (3.46-3.68)	3.63 (3.54-3.72)	3.68 (3.59-3.77)	-0.68 (-1.44-0.08)	.08
Patient experience with self-management	386	4.85 (4.76-4.93)	4.87 (4.80-4.95)	4.78 (4.67-4.88)	4.72 (4.61-4.83)	0.38 (-0.29-1.06)	.26
GP experience with coordination of care around the patient	432	5.92 (5.58-6.27)	7.11 (6.81-7.42)	6.76 (6.34-7.17)	8.18 (7.94-8.42)	-5.28 (-10.64-0.07)	.04

## Discussion

### Summary of Results

There were 290 patients who participated in the intervention group and 392 in the control group. In the intervention group 76/290 (26.2%) of the patients actively used ZWIP. After a follow-up of 12 months, we observed no significant improvement on primary patient outcomes, ADL, IADL, and mental health.

### Strengths and Limitations

The online ZWIP platform was specifically developed for reducing fragmentation of care delivery in older people. Almost half of a frail elderly population without exclusion criteria could be included in the intervention group for using the online ZWIP tool [6]. This is modestly higher than what can be expected in the Dutch context, where 39% persons older than 75 years report having Internet access [22]. This study has two important limitations that can impact results. First, due to the observational nature of the study, comparability between the intervention and the control groups was limited. Despite adjusting for a range of covariates, there may be residual confounding.

Observational, controlled before-after designs are common for complex interventions, where randomized controlled trials (RCTs) are often not appropriate or feasible for evaluation [23]. In the case of ZWIP, contamination between patients would have made individual-level randomization inappropriate. Cluster randomization was not feasible because the level of commitment required from a number of local stakeholders could not be sustained in the control group.

A second limitation was the fact that actual usage of ZWIP was low, even though the implementation of ZWIP was prepared systematically during the development of ZWIP, as this is a structural part of intervention mapping [1,6,7,24,25]. Additionally, implementation strategies were added or adapted when needed during the actual implementation phase. A wide range of implementation strategies were used to encourage uptake; for example, a training program was developed for professionals and an active recruitment phase led to a high participation of older persons. Therefore, low levels of use were attained not because of the lack of, but despite using state of the art implementation techniques. Failure to integrate eHealth interventions in health care is widespread [26], and therefore the low levels of use of these frail older subjects is not surprising. This is especially true for sustained usage of an eHealth intervention [27]. As in other studies [26], further efforts should be focused on improving usability of the intervention, in terms of compatibility for frail older people in chronic disease trajectories [28].

### Future Directions

In addition to further refinement, it is essential to identify those who benefit most from ZWIP and eHealth applications in general. The use of eHealth applications in frail populations could be increased by first identifying frail people with a high likelihood of early adoption of the eHealth intervention, such as people with high computer literacy. Which frail elderly are likely adopters requires further research [26]. Therefore, we plan to perform a quantitative and qualitative evaluation of ZWIP usage as well, going beyond the scope of this paper. We must recognize that in the early stages of evaluation, we take more of an efficacy approach to the evaluation, rather than a pragmatic trial approach. Although the efficacy approach limits generalizability, it allows a thorough investigation of the intervention’s working mechanisms under more controlled, laboratory-like conditions. Such work may also reveal ideal levels of use of ZWIP, as it is possible that communication was already adequate in the case of some patients, making ZWIP usage superfluous. Using both quantitative and qualitative methods in this development phase may elicit remaining barriers and reveal more effective implementation strategies. Only after adapting to this group and proven efficacy is large-scale implementation warranted. Successful wide-scale implementation is a precondition for investigating the effectiveness of eHealth interventions. Otherwise finding no differences between treatment arms cannot be interpreted as a lack of effectiveness. These arguments show that, sufficient time and resources are required to develop, test, and retest new eHealth interventions before finally evaluating their effectiveness in pragmatic trials [29,30].

### Conclusions

Overall, the study confirmed that introducing eHealth interventions in the elderly is a difficult task. Despite using a theory-driven intervention design and state of the art implementation techniques, usage remained low and effectiveness was not observed. Performing a thorough proof of principle study in early adopters may be crucial to improving the use of eHealth interventions in the elderly before evaluating effects on a larger scale.

## Multimedia Appendix 1

Short video of ZWIP in use.

## Abbreviations

ADL: Activities of Daily Living
ANCOVA: analysis of covariance
EASYcare-TOS: EASYcare Two-step Older person Screening
GP: general practitioner
IADL: Instrumental Activities of Daily Living
OHC: Online Health Community
ZWIP: Health and Welfare portal

## References

[1] Robben SH, Huisjes M, van Achterberg T, Zuidema SU, Olde Rikkert MG, Schers HJ, Heinen MM, Melis RJ, ZOWEL NN Study Group (2012). Filling the Gaps in a Fragmented Health Care System: Development of the Health and Welfare Information Portal (ZWIP). JMIR Res Protoc.

[2] Mitnitski AB, Mogilner AJ, Rockwood K (2001). Accumulation of deficits as a proxy measure of aging. ScientificWorldJournal.

[3] Stange KC (2009). The problem of fragmentation and the need for integrative solutions. Ann Fam Med.

[4] Weiner M, Callahan CM, Tierney WM, Overhage JM, Mamlin B, Dexter PR, McDonald CJ (2003). Using information technology to improve the health care of older adults. Ann Intern Med.

[5] van der Eijk M, Faber MJ, Aarts JW, Kremer JA, Munneke M, Bloem BR (2013). Using online health communities to deliver patient-centered care to people with chronic conditions. J Med Internet Res.

[6] Robben SH, Perry M, Huisjes M, van Nieuwenhuijzen L, Schers HJ, van Weel C, Rikkert MG, van Achterberg T, Heinen MM, Melis RJ (2012). Implementation of an innovative web-based conference table for community-dwelling frail older people, their informal caregivers and professionals: a process evaluation. BMC Health Serv Res.

[7] Bartholomew LK, Parcel GS, Kok G, Gottlieb NH, Fernandez ME (2011). Planning health promotion programs: An intervention mapping approach.

[8] Bandura A (2004). Health promotion by social cognitive means. Health Educ Behav.

[9] Bandura A, Ramachandran VS (2012). Self-efficacy. Encyclopedia of Human Behavior, Second Edition.

[10] Prince M (2004). Does Active Learning Work? A Review of the Research. Journal of Engineering Education.

[11] Butterfoss F, Kegler M, Francisco V, Glanz K (2008). Mobilizing organizations for health promotion: theories of organizational change. Health behavior and health education: theory, research and practice.

[12] van Kempen JA, Schers HJ, Jacobs A, Zuidema SU, Ruikes F, Robben SH, Melis RJ, Olde Rikkert MG (2013). Development of an instrument for the identification of frail older people as a target population for integrated care. Br J Gen Pract.

[13] van Kempen JA, Schers HJ, Jacobs A, Zuidema SU, Ruikes F, Robben SH, Melis RJ, Olde Rikkert MG (2013). Development of an instrument for the identification of frail older people as a target population for integrated care. Br J Gen Pract.

[14] Katz S (1983). Assessing self-maintenance: activities of daily living, mobility, and instrumental activities of daily living. J Am Geriatr Soc.

[15] Weinberger M, Samsa GP, Schmader K, Greenberg SM, Carr DB, Wildman DS (1992). Comparing proxy and patients' perceptions of patients' functional status: results from an outpatient geriatric clinic. J Am Geriatr Soc.

[16] Ware JE, Sherbourne CD (1992). The MOS 36-item short-form health survey (SF-36). I. Conceptual framework and item selection. Med Care.

[17] McDowell I, Newell C (2006). Measuring health: a guide to rating scales and questionnaires.

[18] Goldberg SE, Bradshaw LE, Kearney FC, Russell C, Whittamore KH, Foster PE, Mamza J, Gladman JR, Jones RG, Lewis SA, Porock D, Harwood RH, Medical Crises in Older People Study Group (2013). Care in specialist medical and mental health unit compared with standard care for older people with cognitive impairment admitted to general hospital: randomised controlled trial (NIHR TEAM trial). BMJ.

[19] Lutomski JE, Baars MA, van Kempen JA, Buurman BM, den Elzen WP, Jansen AP, Kempen GI, Krabbe PF, Steunenberg B, Steyerberg EW, Olde-Rikkert MG, Melis RJ (2013). Validation of a frailty index from the older persons and informal caregivers survey minimum data set. J Am Geriatr Soc.

[20] Rockwood K, Mitnitski A (2007). Frailty in relation to the accumulation of deficits. J Gerontol A Biol Sci Med Sci.

[21] Teerenstra S, Eldridge S, Graff M, de Hoop E, Borm GF (2012). A simple sample size formula for analysis of covariance in cluster randomized trials. Stat Med.

[22] Statistics Netherlands (2013). Web Magazine.

[23] Craig P, Dieppe P, Macintyre S, Michie S, Nazareth I, Petticrew M, Medical Research Council Guidance (2008). Developing and evaluating complex interventions: the new Medical Research Council guidance. BMJ.

[24] Brendryen H, Johansen A, Nesvåg S, Kok G, Duckert F (2013). Constructing a Theory- and Evidence-Based Treatment Rationale for Complex eHealth Interventions: Development of an Online Alcohol Intervention Using an Intervention Mapping Approach. JMIR Res Protoc.

[25] Vonk Noordegraaf A, Huirne JA, Pittens CA, van Mechelen W, Broerse JE, Brölmann HA, Anema JR (2012). eHealth program to empower patients in returning to normal activities and work after gynecological surgery: intervention mapping as a useful method for development. J Med Internet Res.

[26] Vedel I, Akhlaghpour S, Vaghefi I, Bergman H, Lapointe L (2013). Health information technologies in geriatrics and gerontology: a mixed systematic review. J Am Med Inform Assoc.

[27] Eysenbach G (2005). The law of attrition. J Med Internet Res.

[28] Scandurra I, Sjölinder M (2013). Participatory Design With Seniors: Design of Future Services and Iterative Refinements of Interactive eHealth Services for Old Citizens. Med 2.0.

[29] van Gemert-Pijnen JE, Nijland N, van Limburg M, Ossebaard HC, Kelders SM, Eysenbach G, Seydel ER (2011). A holistic framework to improve the uptake and impact of eHealth technologies. J Med Internet Res.

[30] Makai P, Melis RJ, Olde-Rikkert MG (2014). Technical difficulties and evaluating e-health interventions. JAMA Intern Med.

